# Self-Care Practices among Diabetic Patients in Anand District of Gujarat

**DOI:** 10.1155/2014/743791

**Published:** 2014-02-11

**Authors:** Shyamsundar Jagdish Raithatha, Singh Uday Shankar, Kumar Dinesh

**Affiliations:** Department of Community Medicine, Pramukhswami Medical College, Karamsad, Gujarat 388325, India

## Abstract

*Background*. Diabetes care requires a multipronged approach, wherein the patient has an important role to play. This study was undertaken to explore self-care practices of diabetic patients residing in Anand district of Gujarat. *Methods*. A cross-sectional study, involving 100 diabetic patients, was conducted in 2009-2010. Self-care practices in seven domains of physical activity, dietary practices, medication taking, monitoring of glucose, problem solving, foot care, and psychosocial adjustment were assessed using scores assigned to participants' responses. *Results*. The mean age was 60.9 (SD = 12.2) years and 57% were males. Majority (92%) were Hindus and were consulting private medical practitioners (71%). “Medication taking” was the domain with the best performance score (88.1%) and “problem solving” the worst (11.0%). The “psychosocial adjustment” of the participants was satisfactory (82.5%). Overall mean performance percentage score was 54.41%. Males had better performance scores as compared to females in areas of “physical activity,” “dietary practices,” and “problem solving.” Housewives had poorer performance scores. Total mean performance score was similar for patients on treatment from specialists and general practitioners. *Conclusion*. A self-care education program designed for this region should address the lacunae identified in various domains with a special emphasis on females.

## 1. Introduction

Diabetes, with a global prevalence of 8.3%, affects about 371 million people around the world leading to around 4.8 million deaths every year. Around 80% of the world's diabetic population lives in developing countries [1], India, with a diabetic population of around 63 million [1] ranks second in the list of countries affected by diabetes, with China topping the list with an estimated 92.3 million diabetic patients. Diabetes is a chronic disease, requiring a multipronged approach for its management, wherein the patient has an important role to play. The patients are required to follow certain self-care practices in order to achieve an optimal glycemic control and prevent complications such as neuropathy, nephropathy, and retinopathy. These practices include regular physical activity, appropriate dietary practices, daily foot care practice, compliance with the treatment regimen, and tackling complications such as hypoglycaemic episodes. [2–4] It has been proved that group based educational training programs for the patients in these areas result in improved fasting blood glucose levels, glycated hemoglobin, reduced systolic blood pressure levels, body weight, and the requirement for diabetes medication [5]. In the Indian setting, such educational programs are hardly seen. In order to develop such an educational program, we need to make a baseline assessment of the self-care practices of the patients. It would help us in identifying the lacunae in the patients' practices as well as serve as a benchmark for future comparisons in order to assess the effectiveness of a program. Taking into consideration these issues, a study was designed to explore the self-care practices of the diabetic patients of five villages and two urban slums of Anand district in Gujarat, India.

## 2. Materials and Methods

It was a cross-sectional study carried out between December 2009 and June 2010. The study participants were identified from five villages and two urban slums in the service areas of Charutar Arogya Mandal (CAM). CAM is a not for profit organization involved in provision of health care services in Anand district of Gujarat since 1971. It is involved in community based health care and health promotional activities through 26 villages, 1 town, and 8 health centers in the region. The participants were identified through purposive sampling method with the help of female health workers, Anganwadi workers, and other field staff appointed by CAM. The inclusion criteria for the participants were age ≥20 years, person reporting as suffering from diabetes, resident of Anand district, and a person who is physically and mentally fit to participate in the interview. Exclusion criteria were identified as lower limbs amputated, wheel chair or bed ridden state, and inability to count fingers at 3 metres. As shown in Table 1, the self-care practices among the participants were studied in all the seven domains recommended by the American Association of Diabetes Educators (AADE) known as the “AADE 7 measures of outcome measurement” [6]. This ensured that the content validity of the instrument was taken care of. The psychosocial adjustment (PsA) was judged using the Patient Health Questionnaire-2 (PHQ-2) instrument which is commonly applied to screen for depression [7]. The physical activity (PA) domain was measured using the questions from the Indian Diabetes Risk Score (IDRS) [8]. For the other five domains, questions were designed taking into consideration two readily available instruments and the Indian scenario. The two scales that were referred to were the Self-Care Inventory (SCI) [9] and the Summary of Diabetes Self-Care Activities measure (SDSCA) [10]. The newly designed questionnaire was reviewed by three professionals, namely, a public health expert, a diabetologist, and a social scientist, and their suggestions were utilised for improving the questionnaire thus ensuring consensus validity for the instrument. Moreover the instrument was well structured, the responses were coded, and it was administered in local language. The instrument which was originally designed in English was translated and back translated to and from Gujarati (local) language to ensure appropriateness of the translation. Scores were assigned as per the responses of the participants to each of the questions. Thereafter, the scores of each of the questions in a particular domain were added to get a performance score in that domain. Thus by summing up the scores of all the seven domains, a maximum overall score of 90 was possible. The Mean Performance Score (MPS) calculated as an average of the performance scores of the participants in a domain was treated as the outcome variable. In order to compare the performance of the participants across the seven domains, a Mean Performance Percentage Score (MPPS) was derived for each of the domains, by calculating the percentages for the MPS obtained in each domain with respect to the maximum score possible in that domain. The sample size was calculated with the objective of estimation of the Mean Performance Score (MPS) among the participants using a newly designed survey instrument. The standard deviation (SD = 9.40) in the performance score obtained from the first twenty participants was used as a measure of variance in the score in the population, as the data regarding the variance were not available for the instrument being used for the first time. Taking into consideration an acceptable error of 2 units on either side of the mean and an alpha error of 0.05, the sample size was calculated to be 88 using the software Winpepi [11]. 100 participants were included in the study out of which 50 belonged to the villages and 50 belonged to the slums. An informed written and signed consent for participation in the study was taken from all the participants, in Gujarati (local vernacular) language. Ethical approval for the study was obtained from the Human Research Ethics Committee of Charutar Arogya Mandal (CAM). Data entry was done using Microsoft Excel 2007 and all the entries were double checked with the physical formats for rectification of any possible errors. Data validation checks were introduced during the data entry process to minimize errors during the process of data entry. Data analysis was done using Statistical Package for Social Sciences (SPSS) Version 15. The association of self-care practices in different domains was tested with various demographic, socioeconomic, and diabetes related independent variables using univariate analysis (independent *t*-test, ANOVA, and Pearson's correlation coefficient). One way analysis of variance (ANOVA) was applied for the nominal independent variables with more than two categories, that is, family structure, education, and occupation of self. Post hoc analysis was done using Tukey's honestly significant difference (HSD) test.

## 3. Results and Discussion

### 3.1. Results

**Figure 1:**
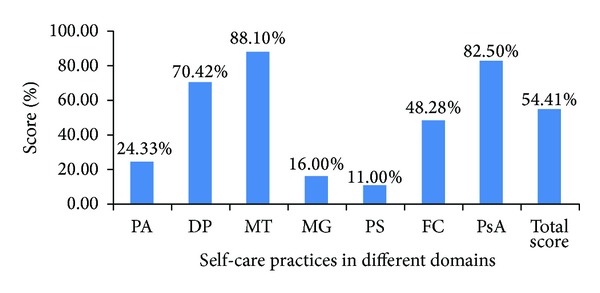
MPPS (mean performance percentage scores) in the seven domains of self-care practices (*n* = 100). PA: physical activity; DP: dietary practices; MT: medication taking; MG: monitoring of glucose; PS: problem solving; FC: foot care; PsA: psychosocial adjustment.

There were 53% male and 47% female participants with a mean age of 60.9 years (SD = 12.2). Among them, 69% had completed at least primary education (7 years of schooling). Majority (92%) belonged to the Hindu religion. 78% of them were married and living with a life partner. 33% of the participants were employed in some form of work, 22% had retired from work, and 45% were housewives. Other sociodemographic characteristics of participants are presented in Table 2. The average duration of diabetes was 8.8 (SD = 7.9) years. Majority (67%) were taking treatment from a physician (MD in medicine) and 27% from a general practitioner with a qualification of MBBS. Most of the participants (71%) were taking treatment from private practitioners. Among the participants, 6%, 11%, and 30% persons reported suffering from eye, cerebrovascular, and cardiac complications, respectively, while none reported renal complications. Only 40% participants had their blood glucose under control as assessed by reviewing their latest blood sugar reports. Other diabetes related features of participants are provided in Table 3. No significant difference was found in the MPSs of urban and rural participants in the study. Medication taking as a domain had the maximum MPPS (88.1%) and problem solving was the domain with the minimum MPPS-11% (Figure 1). The findings in each of the domains are described below.

Physical activity (PA) domain: only forty (40%) participants reported involving in some form of exercise in their leisure time at least 3 days a week. Dietary practices (DP): only 19% of participants reported an intake of at least one serving of raw vegetables or fruits per day “most of the times” or “always.” 48% and 63% of participants reported controlling their intake fat containing foods and sweets, “most of the times” or “always,” respectively. Medication taking (MT): 82% of participants reported that they always took medicines at regular intervals and 68% reported that they had not missed a single dose of medicines in the past one week. Monitoring of glucose: only 16% participants reported self monitoring of blood sugar. Problem solving (PS): only 3% of participants were aware of the medical alert ID card. Only 20% of participants were carrying sugar with them which could be useful for responding to hypoglycemic episodes. Foot care (FC): only 9% of participants examined their feet on a routine basis for any damage to the skin. 12% examined the interior of their foot-wear to look for any thorns or other foreign body. Only 4% of participants were wearing footwear inside the house. Psychosocial adjustment (PsA): 10% of participants were categorized as having depression using the operational definition of PHQ ≥ 3. The MPS for PsA for those having some other chronic illness (4.65) was significantly less (*P* = 0.03) than those not having any other chronic illness (5.23).

The distribution of the MPSs for the six domains (monitoring of glucose excluded) with respect to sex and occupation is shown in Table 4. Males had a better MPS than females in the domains of PA, DP, and PS. The MPS for DP and PS was the highest among those retired from their work, followed by those employed in some form of work and housewives. The MPS for PA was the highest among those currently employed followed by those retired and the least among the housewives. Participants staying with a life partner had a greater MPS (2.33) in the domain of PA as compared to those staying single (1.68, *P* = 0.03). Other variables like education, family structure, family size, income, and addictions were not significantly associated with differences in MPS across various domains. Similarly variables related to diabetes profile like family history, duration since diagnosis, type of treatment, and presence of complications were not associated with significant differences in MPS across various domains. Total MPS was higher for patients on treatment from a specialist—MD physician (50.04)—as compared to those on treatment from a general practitioner—MBBS doctor (46.59)—with a *P* value of 0.09.

### 3.2. Discussion

This was a cross-sectional study designed to explore the self-care practices among diabetic patients by calculating the MPS for each of the seven domains of self-care. The reasons for not finding a difference in the MPS of urban and rural participants could be similar treatment seeking behaviors of urban and rural participants with most of them consulting private practitioners and that most of the rural participants belonged to a higher socioeconomic background. Males had a better MPS in the domain of PA as compared to females which could be due to several reasons. Firstly, most of the females (45 out of 47) were housewives and 28 (60%) out of them belonged to families with a monthly income of 15,000 and above, where a maid could be easily appointed for carrying out the routine household work. Secondly, only 23 (49%) females were involved in some exercise as compared to 33 (62%) males. The overall control of fat containing foods (48%) and sweets (63%) in diet was better than that seen in the study conducted in the resettlement colony of Chandigarh in 1998, where it was found that only 18.3% of the 60 participants were avoiding sweets and fatty foods [12]. This could be because of a different geographical and sociocultural environment and probably an increased level of awareness over time. Good compliance with MT was reported in this study. Similar findings were also reported in the study conducted in the resettlement colony of Chandigarh in 1998 [12] and in Trichy in 2002 [13] where the compliance rates with medicines were 62.9% and 75%, respectively. FC practices were explored in quite detail based on nine different questions. Wearing footwear inside the house, which is a common practice in the western countries, was very rare in this study. Similar findings were observed in a multicentre study conducted in India in 2005, wherein only 3% of the participants were wearing footwear inside the house [14]. However in a study conducted in Mumbai, 55% of the participants reported wearing footwear inside the house which could be because of different sociocultural background of the participants, the details of which have not been mentioned in the study [15]. In the Chandigarh study, FC was done by 63.3% of the participants through regular washing [12]. In our study, 82% reported washing their feet with soap and water on a daily basis. In the other areas of FC, great deal of improvement in practices is required. In the domain of PsA, majority of the participants appeared to have adjusted well to their disease status. The lower MPS found among those having other coexisting chronic disease as compared to those not having the disease is expected, as the presence of other coexisting illnesses puts an additional burden on the person as well as the family in terms of social, economic, and psychological aspects.

Thus an instrument was designed and used to comprehensively measure the self-care practices among diabetic patients. Very few instruments are available to measure all the domains of self-care practices. One such instrument called Summary of Diabetes Self-Care Activities (SDSCA) is available. It tests the self-care practices in five areas, namely, diet, exercise, blood sugar testing, medications, and foot care during the previous week. This instrument shows good internal and test-retest reliability and can be generalized to various patient populations with diabetes [10]. One other instrument called the Diabetes Self-Care Inventory (SCI) has been designed especially for type 1 diabetic patients and it tests self-care in four areas, namely, blood glucose testing and monitoring, insulin and food regulation, exercise, and emergency precautions (e.g., carrying sugar to treat reactions) [9]. However these two have not been tested in the Indian setting and they miss out important areas such as PS and PsA, which were covered in this study. The survey instrument is a new one. Although several aspects of the validity of the instrument have been taken care of while designing it, it should be further tested for construct validity by comparing it with the glycemic control of the participants.

## 4. Conclusions

The deficiencies identified in the self-care practices suggest a dire need to develop and integrate diabetes self-care education programs in routine clinical practice. As most of the diabetic patients consult private practitioners for treatment, the private practitioners should be involved in the whole process of planning, implementation, and evaluation of the program. Any newly designed diabetes self-care education program envisaged for this region should concentrate particularly on areas like problem solving skills, foot care practices, dietary practices, and physical activity. Moreover it should have a special focus on females as their practices were found to be deficient in several domains.

## Figures and Tables

**Table 1:** List of domains of self-care practices, areas explored in each of the domains, and maximum score possible in each of the domains.

Sr. no.	Domains of self-care activities	Areas explored	Maximum score possible
1	PA	Physical activity at work, at home, and in leisure time	9
2	DP	Regularity in taking meals, skipping meals, avoiding fat rich foods in diet, avoiding sugar rich foods in diet, and intake of raw vegetables and fruits in diet.	19
3	MT	Regularity in taking medicines, skipping a dose of medicine in last one week	10
4	MG	Use of glucometer or uristicks for monitoring glucose control	1
5	PS	Carrying a medical alert identification card, carrying sugar rich foods for responding to hypoglycemic episodes	6
6	FC	Daily examination of feet, daily washing feet with soap and water, cleaning and drying space between the toes, daily examination of footwear, soaking the feet, Wearing footwear inside the house, preference for treating a corn/callus on feet, checking temperature of water before applying on feet, and wearing socks with footwear	39
7	PsA	2 questions based on PHQ 2 [8]	6

	Total score		90

PA: physical activity; DP: dietary practices; MT: medication taking; MG: monitoring of glucose; PS: problem solving; FC: foot care; PsA: psychosocial adjustment.

**Table 2:** Sociodemographic profile of the study participants.

Item	Value
Total number of participants	100
Sex	
Males	53
Females	47
Location	
Urban	50
Rural	50
Religion	
Hindu	92
Muslim	4
Christian	2
Other	2
Marital status	
Married—living with a life partner	78
Single—widowed or divorcee	22
Family structure	
Joint	18
Nuclear	45
Three-generation family	37
Mean family size (SD)	4.79 (2.3)
Median family size	5
Addiction (percent)	
None	73 (70.9)
Smoker	12 (11.7)
Oral tobacco	16 (15.5)
Alcohol	0
Other	2 (1.9)
Education	
Illiterate	10
Primary/literate	21
Middle/secondary	43
High school	4
Intermediate/post-high-school diploma	8
Graduate and above	14
Occupation	
Currently working	33
Retired	22
Housewife	45
Monthly family income	
0–2500	4
2500–5000	5
5000–10000	15
10000–15000	19
15000–20000	20
≥20000	37

**Table 3:** Diabetic profile of the study participants.

Item	Value
Mean duration of diabetes in years (95% C.I.)	8.75 (7.18–10.31)
History of diabetes in first degree relatives	
Present	44
Absent	56
Other coexisting illnesses	
Hypertension (%)	45 (40.5)
Ischemic heart disease (%)	10 (9)
Osteoarthritis (%)	2 (1.8)
Others (%) (polio, thyroid)	2 (1.8)
None (%)	52 (46.9)
Qualification of the doctor being consulted	
MBBS—general practitioner	27
MD—physician	67
Endocrinologist/diabetologist	1
Ayurvedic—practitioner	1
Homeopathy—practitioner	1
Other	3
Type of treatment facility being consulted	
Private practitioner/clinic	71
Multispecialty hospital	0
Medical college hospital	9
Government hospital-PHC/CHC/district hospital	2
UHTC	10
RHTC	3
Other	5
Type of treatment being undertaken	
Oral drugs prescribed by a physician (%)	94 (87)
Insulin (%)	6 (5.6)
Ayurvedic medicines (%)	8 (7.4)
Homeopathic medicines (%)	0
Others (%)	0
History of complications*	
Eye	
Present (doctor said that eyes have been affected)	6
Not tested	61
Kidney	0
Cardiovascular	30
Cerebrovascular	11
Glycemic control^$^	
Controlled (%)	40
Not controlled (%)	52
Not available	8

PHC: Primary Health Center; CHC: Community Health Center; UHTC: Urban Health Training Center; RHTC: Rural Health Training Center.

*As per the history given by the patient—patient self-report. ^$^Glycemic control: when the information obtained for glucose control satisfied all of the three criteria, that is, FBS < 126 mg/dL, PP2BS < 180 mg/dL, RBS < 200 mg/dL, and Hba_1_c < 7 gm%, it would be considered as “controlled.” If any one of the criteria is not met, it would be considered as “not controlled.”

**Table 4:** MPS in different domains with respect to important independent variables.

Variable	MPS in different domains (95% confidence intervals)					
	PA	DP	MT	PS	FC	PsA
Overall MPS	2.19 (1.93–2.44)	13.38 (12.87–13.88)	8.81 (8.37–9.25)	0.66 (0.39–0.93)	18.83 (15.58–20.08)	4.95 (4.68–5.22)
Sex						
Male (53)	2.45 (2.09–2.81)	13.96 (13.40–14.52)	8.51 (7.84–9.18)	0.98 (0.53–1.43)	19.81 (18.02–21.61)	5.04 (4.66–5.42)
Female (47)	1.89 (1.54–2.25)	12.72 (11.86–13.58)	9.15 (8.6–9.7)	0.30 (0.04–0.56)	17.72 (15.98–19.47)	4.85 (4.47–5.23)
*P* value	0.03	0.01	0.14	0.01	0.099	0.49
Occupation*						
Retired (45)	2.14 (1.58–2.69)	14.09 (13.24–14.93)	8.73 (7.64–9.81)	1.23 (0.34–2.11)	19.86 (16.53–23.19)	5.04 (4.40–5.69)
Currently working (33)	2.73 (2.27–3.18)	14.03 (13.25–14.81)	8.45 (7.59–9.31)	0.76 (0.31–1.21)	20.21 (18.08–22.34)	5.09 (4.62–5.56)
Housewife (22)	1.82 (1.47–2.17)	12.56 (11.70–13.41)	9.11 (8.54–9.68)	0.31 (0.04–0.58)	17.31 (15.61–19.01)	4.8 (4.41–5.19)
*P* value	0.007	0.01	0.43	0.03	0.09	0.60

*One way ANOVa was applied for testing the significance.

PS: problem solving; PA: physical activity; DP: dietary practices; MT: medication taking; FC: foot care; PsA: psychosocial adjustment.
